# The efficacy of acupuncture with Lingguibafa acupoint selection in the treatment of insomnia: A PRISMA-compliant meta-analysis

**DOI:** 10.1097/MD.0000000000031515

**Published:** 2022-10-28

**Authors:** Haiyang Ji, Ke Zhang, Yunqiong Lu, Xiehe Kong, Xiaopeng Ma

**Affiliations:** a Yueyang Hospital of Integrated Traditional Chinese and Western Medicine, Shanghai University of Traditional Chinese Medicine, Shanghai, China; b The First Clinical Medical College, Shandong University of Traditional Chinese Medicine, Jinan Shandong, China; c Shanghai Research Institute of Acupuncture and Meridian, Shanghai, China.

**Keywords:** analysis, randomized controlled trial, dialectical acupoint selection, insomnia, acupuncture, meta, Lingguibafa acupoint selection

## Abstract

As a major public health problem, insomnia has garnered much attention. Acupuncture, with dialectical acupoint selection (DAS), has been proved to be effective for insomnia, but there is no evidence of evidence-based medicine to prove the efficacy of Lingguibafa acupoint selection (LAS) for insomnia. This study is to evaluate the clinical efficacy of acupuncture with LAS in patient with insomnia.

**Methods:**

The PubMed, Web of Science, Embase, Cochrane Library, China National Knowledge Infrastructure, Weipu Database for Chinese Technical Periodicals, Chinese Biomedical Database, and Wanfang Database were systematically searched from the inception dates to December 18, 2021. Randomized controlled trials of acupuncture with LAS versus acupuncture with DAS or acupuncture with LAS plus DAS versus acupuncture with DAS in patient with insomnia were included. Two review authors independently performed the data extraction and assessed study quality. A meta-analysis was performed using random-effects models to calculate relative risk and weighted mean difference for categorical and continuous variables, respectively. The primary efficacy outcome was improvement on Pittsburgh Sleep Quality Index (PSQI). Secondary outcomes included insomnia severity index, Epworth sleepiness scale, Hamilton anxiety scale, Hamilton Depression Scale (HAMD), and total effective rate.

**Results:**

A total of 7 trials with 468 patients fulfilled the selection criteria. The pooled results indicated that acupuncture with LAS plus DAS could reduce PSQI score by 2.08 (1.60 after excluding heterogeneity) compared to acupuncture with DAS. Acupuncture with LAS plus DAS had high reliability in sleep quality, sleep latency, sleep time and daytime function, but showed no significant effect on sleep disorder and hypnotic drug. For total effective rate, acupuncture with LAS plus DAS or acupuncture with LAS was better than acupuncture with DAS, but the conclusion was less credible.

**Conclusion:**

Acupuncture with LAS is significantly associated with improvements in several sleep parameters, primarily evident on the PSQI score. Nevertheless, considering the poor methodological quality, trials employing appropriate randomization concealment and blinding based on a larger sample size are needed in the future.

## 1. Introduction

Insomnia, as a major public health problem, has garnered much attention. It was characterized by difficulty initiating or maintaining sleep, with a variety of symptoms such as fatigue, depression, irritability, and anxiety.^[1,2]^ The incidence rate of insomnia is approximately 10% to 20% worldwide and 15% in China, of which more than half are chronic insomnia that occurs 3 times a week and lasts for more than 1 month.^[3,4]^ Insomnia affects the quality of life, physical and mental health of patients, and is closely related to the incidence of hypertension, atherosclerosis, gastroesophageal reflux and some other diseases.^[5–8]^

As an essential branch of traditional Chinese medicine (TCM), acupuncture is widely used in the treatment of insomnia^[9]^ since a proportion of patient still suffered insomnia despite treatment with hypnotic drug. Most of time, acupuncture points for insomnia are chosen according to the patient’s symptoms, which is called dialectical acupoint selection (DAS). Lingguibafa acupoint selection (LAS), an idea of time medicine in TCM, is to select acupoints according to the time in 2 hours. TCM Chinese doctors believed that there are 8 acupoints in the human body that link the qi and blood of the whole body, and the state of these 8 points has a time pattern, according to which the stimulation effect is more significant. Previous meta-analysis^[10]^ has suggested that acupuncture is effective in the treatment of insomnia but there is no evidence-based evidence for acupuncture with LAS. This study aimed to evaluate the clinical efficacy of acupuncture with LAS in patient with insomnia, and therefore provide a basis for clinical decision-making.

## 2. Materials and methods

### 2.1. Protocol and registration

This study was registered on INPLASY (INPLASY202240152), and conducted based on the Preferred Reporting Items for Systematic Reviews and Meta Analyses (PRISMA) statement guidelines.

### 2.2. Databases search strategy

We searched PubMed, Web of Science, Embase, Cochrane Library, China National Knowledge Infrastructure, Weipu database for Chinese technical periodicals, Chinese biomedical database, and Wanfang Database from the establishment of the database up to December 18, 2021. The Chinese search terms were primarily “insomnia” “early awakening” “sleep disorder” “difficulty falling asleep” AND “Lingguibafa” “time acupuncture.” The English search terms were as follows: “insomnia” “early awakening” “disorders of initiating and maintaining sleep” “sleep initiation and maintenance orders” “sleep initiation and maintenance orders” AND “Lingguibafa” “Lingui Bafa” “Linggui 8 methods” “8 methods of intelligent turtle.”

### 2.3. Inclusion criteria

1. Participants were diagnosed with insomnia;
2. Trials of acupuncture with LAS versus acupuncture with dialectical acupoint selection (DAS) or acupuncture with LAS plus DAS versus acupuncture with DAS;
3. The main outcome was PSQI;
4. The Secondary outcome was insomnia severity index, Epworth sleepiness scale, Hamilton anxiety scale, HAMD and total effective rate;
5. The language is limited to English and Chinese.

### 2.4. Exclusion criteria

1. Duplicate literature;
2. Animal experiment, protocol, review, conference papers, case reports and experience reports;
3. The trial design is not rigorous.

### 2.5. Study selection and data extraction

HYJ and KZ will independently search all databases, and use NoteExpress 3.3.0 to exclude duplicate literature. Then they will read the title, abstract and full text to determine the qualified trials. YQL and KZ will independently extract date including trials details (title, authors, journal, publication time, method of randomization and blinding), participants characteristics (age, duration, of disease, diagnostic criteria and sample size), interventions and controls, main outcome and secondary outcome. If the information is incomplete, we will contact author by e-mail. Any disagreement will be resolved through discussion with a third reviewer (XPM).

### 2.6. Risk of bias assessment

HYJ and KZ will independently assess the risk of bias of included trials according to the Cochrane Handbook for Systematic Review of Interventions. According to the criteria, each item will be classified as high, low and unclear. Any disagreement will be resolved through discussion with XPM.

### 2.7. Statistical analysis and publication bias

The statistical analysis was carried out by using the statistical software RveMan 5.4 (Nordic Cochrane Center, The Cochrane Collaboration, Copenhagen, Denmark) The random-effects model was used whether the heterogeneity is significant or not. The relative risk was used for the dichotomous data, the weighted mean difference (WMD) for the continuous data, and the 95% confidence interval (95% CI) for confidence level. The sensitivity analysis should be performed to assess the reliability of the meta-analysis by omitting each trial and reevaluating. The publication bias was analyzed by Egger test in Stata 16 statistical analysis software (StataCorp LP, College Station, TX).

## 3. Result

### 3.1. Literature search results

**Figure 1.**
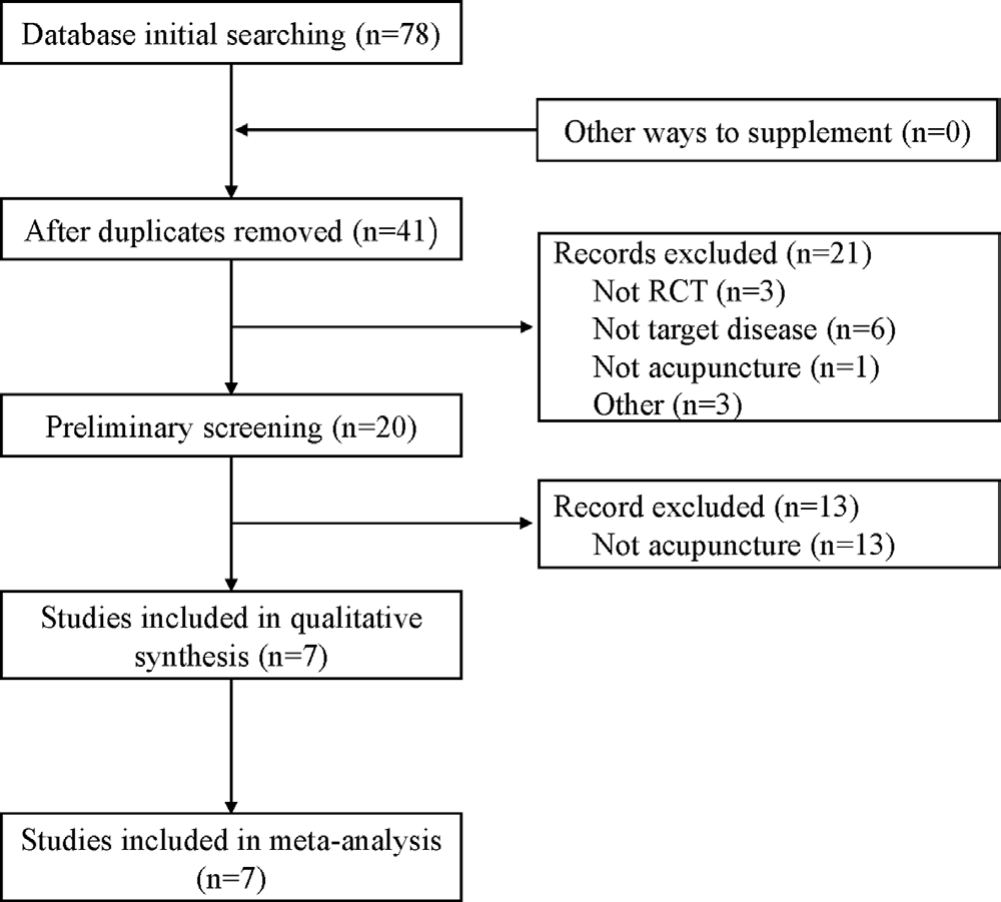
Flow chart of literature retrieval.

From the searches for published randomized controlled trials, 78 records [China National Knowledge Infrastructure (n = 32), WanFang Database (n = 20), Chinese biomedical database (n = 27), Weipu database for Chinese technical periodicals (n = 1)] were identified, and 7^[11–17]^ met the criteria. (Fig.1)

### 3.2. Basic characteristics and quality evaluation of included trials

Seven trials^[11–17]^ with a total of 468 patients were included in meta-analysis, all of which were published in Chinese. From them, 30 cases received acupuncture with LAS, 220 cases received acupuncture with LAS plus DAS, and 218 cases received acupuncture with DAS. The basic characteristics of the included trials are shown in Table 1.

**Table1:** Basic characteristics of included trials.

Trial	Patients (cases) Treatment Group/ Controlled Group	Interventions Treatment Group/ Controlled Group	Course (days)	Main outcome	Source of efficacy evaluation
Li 2010	30/ 30	LAS/ DAS	21	PSQI	①
Cao 2013	30/ 30	LAS + DAS/ DAS	20	PSQI	②
Huang 2015	30/ 30	LAS + ASD/ DAS	28	PSQI	②
Wu 2014	25/ 25	LAS + DAS/ DAS	20	PSQI	①
Yan 2008	30/ 30	LAS + DAS/ DAS	18	PSQI、SRSS、HAMA	④
Wen 2011	45/ 43	LAS + DAS/ DAS	12	PSQI	③
Yao 2016	30/ 30	LAS + DAS/ DAS	20	PSQI	④

Notes:①《Evaluation criteria for clinical efficacy of new traditional Chinese medicine drugs》；②WHO sleep rate test；③《Handbook of mental health assessment scale》;④:Not mentioned.

PSQI = iThe Pittsburgh Sleep Quality Index, HAMA = Hamilton Anxiety Scale.

### 3.3. Bias risk assessment results

**Figure 2.**
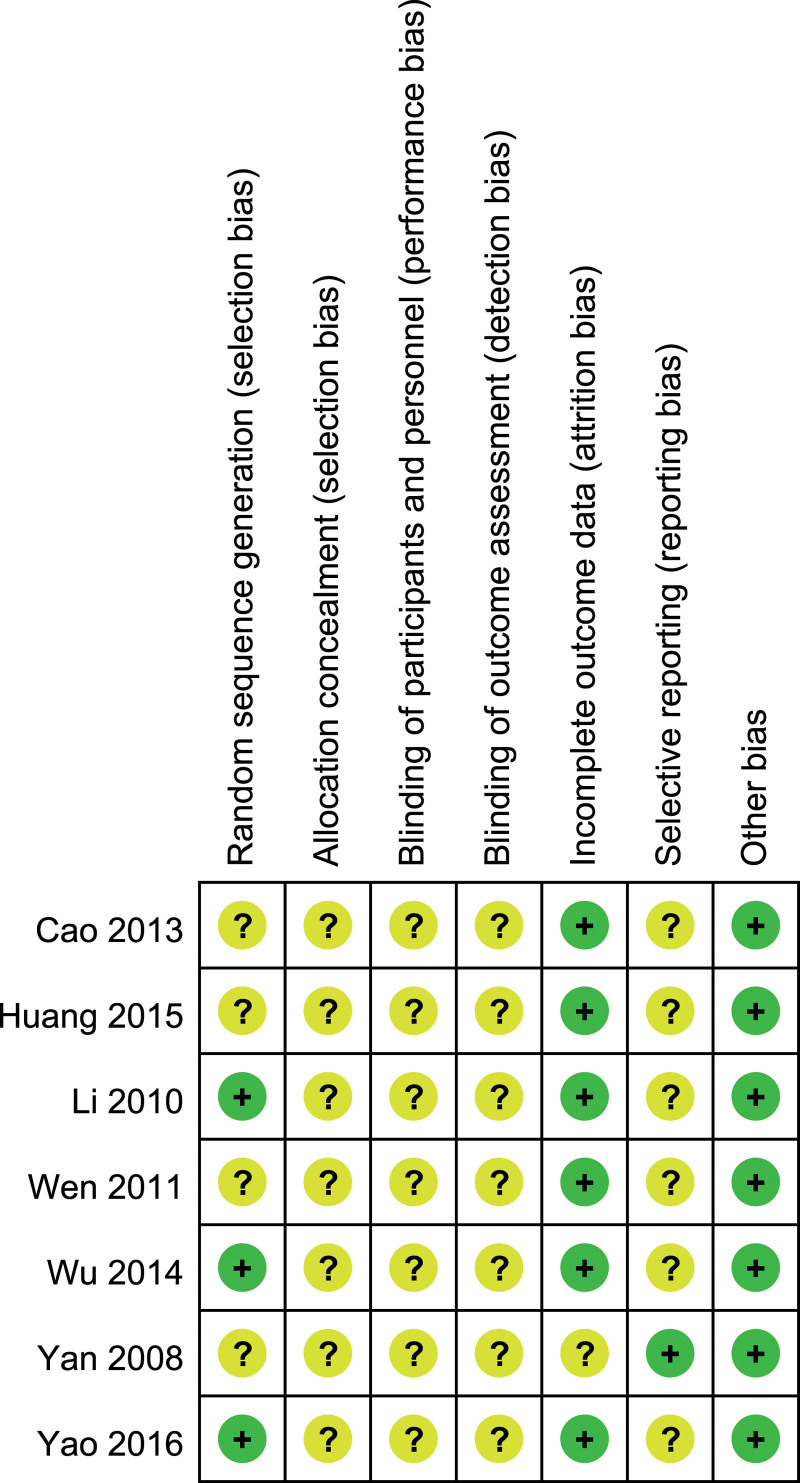
Risk of bias summary.

**Figure 3.**
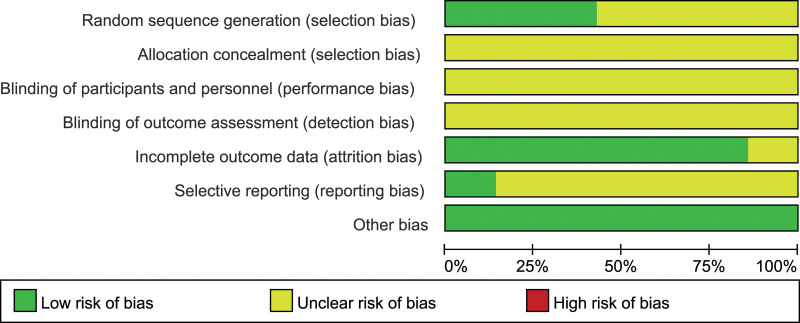
Risk of bias graph.

Seven trials^[11–17]^ had moderate risk of bias: all trials^[11–17]^ used randomized method and 3 of them^[11,13,16]^ described the details of randomization; 2^[11,16]^ reported the follow-up results; allocation concealment was not described in all 7 trials^[11–17]^ (Figs. 2 and 3).

## 4. Meta-analysis results

### 4.1. PSQI total score

**Figure 4.**
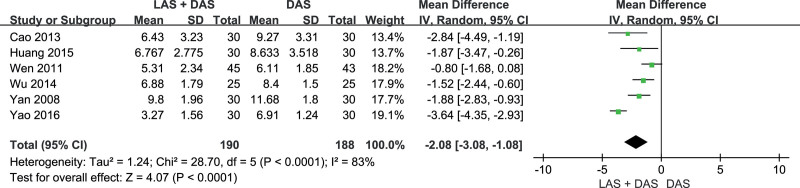
PSQI score. PSQI = The Pittsburgh Sleep Quality Index.

**Figure 5.**
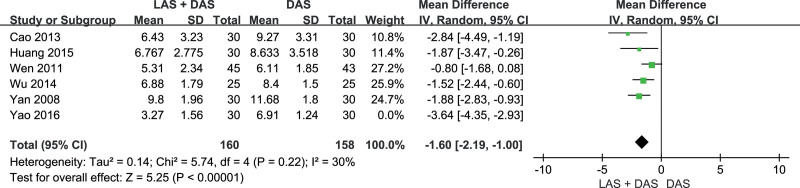
PSQI score (Excluded Yao’s trial). PSQI = The Pittsburgh Sleep Quality Index.

Seven trials^[11–17]^ reported PSQI scores (acupuncture with LAS plus DAS group n = 220, acupuncture with LAS group n = 30, acupuncture with DAS group n = 218). Acupuncture with LAS plus DAS could reduce PSQI score significantly (WMD = –2.08, 95% CI [–3.08, –1.08], *P* < .01) compared to acupuncture with DAS, but the test for heterogeneity was significant (*P* < .01, *I*^2^ = 83%). After excluding Yao trial,^[11]^ there was no obvious heterogeneity (*P* < .22, *I^2^* = 30%), and the result was the same as before (WMD = –1.60, 95% CI [–2.19, –1.00], *P* < .01) (Figs.4 and 5).

### 4.2. Analysis of PSQI indicators

#### 4.2.1. Sleep quality, sleep latency and sleep time.

**Figure 6.**
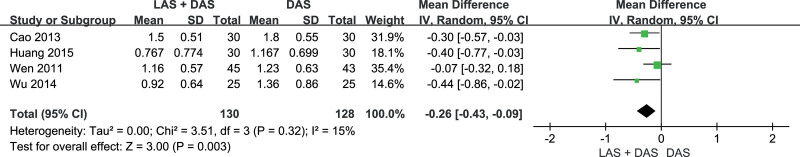
Sleep quality.

**Figure 7.**
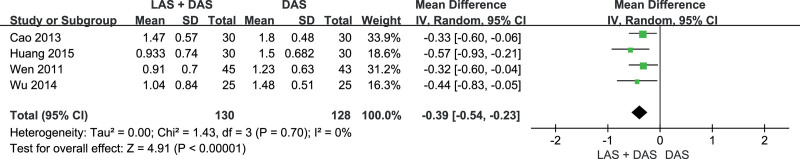
Sleep latency.

**Figure 8.**
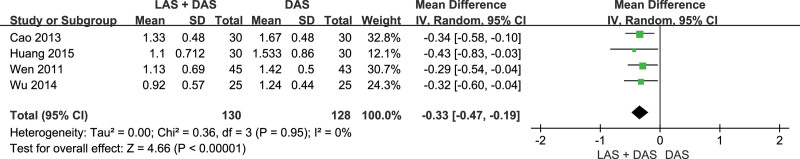
Sleep time.

Five trials^[12–16]^ reported the PSQI scores of sleep quality, sleep latency and sleep time (acupuncture with LAS plus DAS group n = 130, acupuncture with LAS group n = 30, acupuncture with DAS group n = 158). There heterogeneity was not statistically significant for these 3 outcomes (*P* =.32, 0.70, 0.95, *I*^2^ = 15%, 0%, 0%). The score was lower in the acupuncture with LAS plus DAS group than in the acupuncture with DAS group (WMD = –0.26, –0.39 –0.33, 95% CI = [–0.43, –0.09], [–0.54, –0.23], [–0.47, –0.19], *P* < .01) (Figs. 6–8).

#### 4.2.2. Sleep efficiency.

**Figure 9.**
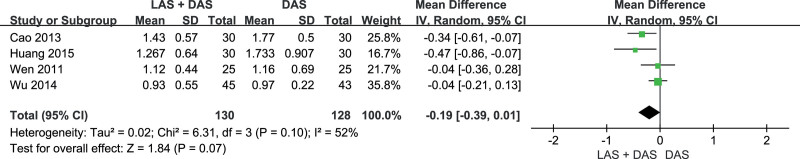
Sleep efficiency.

**Figure 10.**
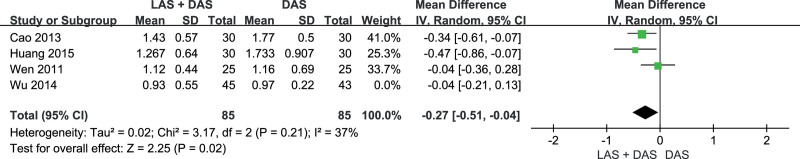
Sleep efficiency (Excluded Wen’s trial).

Five trials^[12–16]^ reported sleep efficiency score (acupuncture with LAS plus DAS group n = 130, acupuncture with LAS group n = 30, acupuncture with DAS group n = 158). No significant difference was found between the 2 groups (WMD = –0.19, 95% CI [–0.39, 0.01], *P* = .07) and the heterogeneity was statistically significant (*P* = .10, *I^2^* = 52%). After excluding Wen trial,^[15]^ the heterogeneity was significantly reduced (*P* = .21, *I^2^* = 37%), and we found a improvement with acupuncture with LAS compared to acupuncture with DAS (WMD = –0.27, 95% CI [–0.51, –0.04], *P* =.02) (Figs. 9 and 10).

#### 4.2.3. Daytime function.

**Figure 11.**
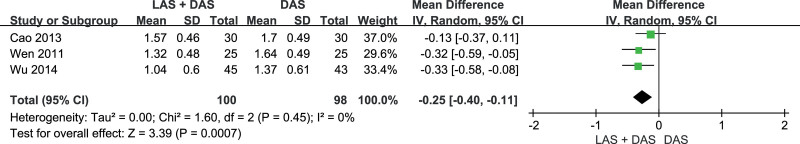
Daytime function.

Four trials^[13–16]^ reported daytime functional scores (acupuncture with LAS plus DAS group n = 100, acupuncture with LAS group n = 30, acupuncture with DAS group n = 128). Acupuncture with LAS plus DAS significantly improved daytime function (WMD = –0.25, 95% CI [–0.40, –0.11], *P* < .01) compared to acupuncture with DAS, and the heterogeneity was not significant (*P* =.45, *I^2^* = 0%) (Fig. 11).

#### 4.2.4. Sleep disorder.

**Figure 12.**
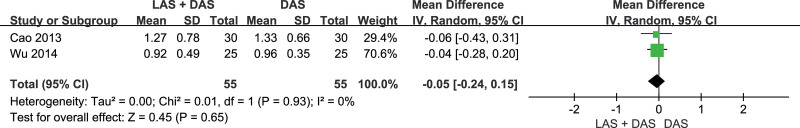
Sleep disorder.

Three trials^[13,14,16]^ reported sleep disorder scores (acupuncture with LAS plus DAS group n = 55, acupuncture with LAS group n = 30, acupuncture with DAS group n = 85). No significant difference was found between the 2 groups (WMD = –0.05 95% CI [–0.24, 0.15], *P* < .65), and heterogeneity was not significant (*P* =.93, *I^2^* = 0%) (Fig.12).

#### 4.2.5. Hypnotic drug.

Two trials^[13,16]^ reported hypnotic drug scores (acupuncture with LAS plus DAS group n = 25, acupuncture with LAS group n = 30, acupuncture with DAS group n = 55). Wu trial suggested that acupuncture with LAS plus DAS did not reduce hypnotic drug score (95% CI [–0.20, 0.36]) compared to acupuncture with DAS, while Li’s trial indicated that acupuncture with LAS reduced hypnotic drug score (95% CI [–0.80, –0.14]) compared to acupuncture with DAS.

### 4.3. Total effective rate

Seven trials^[11–17]^ reported the total effective rate: 2/7^[13,16]^ referred to Evaluation Criteria for Clinical Efficacy of New Traditional Chinese Medicine Drugs, 2/7^[12,14]^ to WHO Sleep Rate Test, 1/7^[15]^ to Handbook of Mental Health Assessment Scale, and 2/7^[11,17]^ did not clearly describe the source of efficacy evaluation. Seven rials^[11–17]^ suggested that, compared to acupuncture with DAS, acupuncture with LAS or acupuncture with LAS plus DAS had better efficacy, but the confidence intervals were wide, indicating that the conclusions were less credible (Table 2).

**Table 2:** Summary of total effective rate of included trials.

Trial	Source of efficacy evaluation	Treatment group		Controlled group		95% CI
		Effective	Total	Ineffective	Total	
Li 2010	①	28	30	25	30	1.12 [0.93, 1.35]
Cao 2013	②	26	30	23	30	1.13 [0.89, 1.44]
Huang 2015	②	28	30	25	30	1.12 [0.93, 1.35]
Wu 2014	①	23	25	17	25	1.35 [1.01, 1.81]
Yan 2008	④	27	30	23	30	1.17 [0.93, 1.48]
Wen 2011	③	41	45	37	43	1.06 [0.91, 1.23]
Yao 2016	④	29	30	25	30	1.16 [0.98, 1.38]

Notes: ① 《Evaluation criteria for clinical efficacy of new traditional Chinese medicine drugs》; ②WHO sleep rate test; ③《Handbook of mental health assessment scale》; ④: Not mentioned.

### 4.4. Summary of each indicators of Li’s trial

Seven trials^[11–17]^ included in this meta-analysis, 6/7^[11–15,17]^ used acupuncture with LAS plus DAS as the treatment group and 1/7^[16]^ used acupuncture with LAS. Li’s trial^[16]^ reported all the outcomes and the effect of acupuncture with LAS alone was the same as that of acupuncture with LAS plus DAS, except for hypnotic drug (Table 3).

**Table 3:** Summary of each result of Li’s trial.

Outcomes	Treatment group			Controlled group			95% CI
	Mean	SD	Total	Mean	SD	Total	
PSQI total score	4.01	4.87	30	7.32	5.88	30	–3.31 [–6.04, –0.58]
Sleep quality	0.83	0.5	30	1.2	0.72	30	–0.37 [–0.66, –0.08]
Sleep latency	1.06	0.78	30	1.62	0.89	30	–0.56 [–0.98, –0.14]
Sleep time	0.63	0.55	30	1.07	0.98	30	–0.44 [–0.84, –0.04]
Sleep efficiency	0.62	0.57	30	1.09	0.73	30	–0.47 [–0.80, –0.14]
Daytime function	0.52	0.31	30	0.99	0.68	30	–0.47 [–0.74, –0.20]
Sleep disorder	0.84	0.68	30	1.01	0.76	30	–0.17 [–0.53, 0.19]
Hypnotic drug	0.49	0.31	30	0.96	0.86	30	–0.47 [–0.80, –0.14]
	Effective	Total	Ineffective	Total	95% CI		
Total effective rate	28	30	25	30	1.12 [0.93, 1.35]		

PSQI = The Pittsburgh Sleep Quality Index.

### 4.5. Sensitivity analysis

The sensitivity analysis was performed to assess the reliability of the meta-analysis by omitting each trial and reevaluating. The meta-analysis results, except for PSQI and sleep efficiency, had no significant change, which indicated the results were relatively reliable. For PSQI score and sleep efficiency, the heterogeneity results changed significantly, but 95% CI was relatively stable (Table 4).

**Table 4:** Summary of Sensitivity analysis.

Outcomes	Omitted trial	95% confidence interval	Heterogeneity
PSQI score	NO	–2.08 [–3.08, –1.08], *P* < .01	*P* < .01, *I*^2^ = 83%
	Cao 2013	–1.96 [–0.39, –0.84], *P* < .01	*P* < .01, *I*^2^ = 86%
	Huang 2015	–2.12 [–3.26, –0.98], *P* < .01	*P* < .01, *I*^2^ = 86%
	Wu 2014	–2.20 [–3.41, –1.00], *P* < .01	*P* < .01, *I*^2^ = 85%
	Yan 2008	–2.13 [–3.37, –.088], *P* < .01	*P* < .01, *I*^2^ = 86%
	Wen 2011	–2.37 [–3.35, –1.40], *P* < .01	*P* < .01, *I*^2^ = 76%
	Yao 2016	–1.60 [–2.19, –1.00], *P* < .01	*P* = .22, *I*^2^ = 30%
Sleep quality	NO	–0.26 [–0.43, –0.09], *P* < .01	*P* = .32, *I*^2^ = 15%
	Cao 2013	–0.26 [–0.51, –0.01], *P* = .04	*P* = .19, *I*^2^ = 40%
	Huang 2015	–0.23 [–0.43, –0.03], *P* = .03	*P* = .25, *I*^2^ = 28%
	Wu 2014	–0.23 [–0.42, –0.04], *P* = .02	*P* = .27, *I*^2^ = 23%
	Wen 2011	–0.36 [–0.55, –0.16], *P* < .01	*P* = .83, *I*^2^ = 0%
Sleep latency	NO	–0.39 [–0.54, –0.23], *P* < .01	*P* = .70, *I*^2^ = 0%
	Cao 2013	–0.42 [–0.61, –0.23], *P* < .01	*P* = .56, *I*^2^ = 0%
	Huang 2015	–0.35 [–0.52, –0.18], *P* < .01	*P* = .87, *I*^2^ = 0%
	Wu 2014	–0.38 [–0.55, –0.21], *P* < .01	*P* = .51, *I*^2^ = 0%
	Wen 2011	–0.42 [–0.51, –0.23], *P* < .01	*P* = .58, *I*^2^ = 0%
Sleep time	NO	–0.33 [–0.74, –0.19], *P* < .01	*P* = .95, *I*^2^ = 0%
	Cao 2013	–0.33 [–0.50, –0.16], *P* < .01	*P* = .84, *I*^2^ = 0%
	Huang 2015	–0.32 [–0.74, –0.17], *P* < .01	*P* = .96, *I*^2^ = 0%
	Wu 2014	–0.33 [–0.49, –0.17], *P* < .01	*P* = .84, *I*^2^ = 0%
	Wen 2011	–0.35 [–0.52, –0.18], *P* < .01	*P* = .90, I^2^ = 0%
Sleep efficiency	NO	–0.19 [–0.39, 0.01], *P* = .07	*P* = .10, *I*^2^ = 52%
	Cao 2013	–0.13 [–0.36, 0.19], *P* = .25	*P* = .15, *I*^2^ = 48%
	Huang 2015	–0.13 [–0.32, 0.06], *P* = .18	*P* = .17, *I*^2^ = 44%
	Wu 2014	–0.27 [–0.51, –0.04], *P* = .02	*P* = .21, *I*^2^ = 37%
	Wen 2011	–0.24 [–0.51, 0.02], *P* = .07	*P* = .06, *I*^2^ = 65%
Daytime function	NO	–0.25 [–0.40, –0.11], P < .01	P = .45, I2 = 0%
	Cao 2013	–0.33 [–0.51, –0.14], *P* < .01	*P* = .96, *I*^2^ = 0%
	Wu 2014	–0.22 [–0.40, –0.03], *P* = .02	*P* = .30, *I*^2^ = 6%
	Wen 2011	–0.23 [–0.42, –0.03], *P* = .02	*P* = .26, *I*^2^ = 21%
Sleep disorder	NO	–0.05 [–0.24, 0.15], *P* = .65	*P* = .93, *I*^2^ = 0%
	Cao 2013	–0.04 [–0.28, 0.20], *P* = .74	–
	Wu 2014	–0.06 [–0.43, 0.31], *P* = .75	–

PSQI = The Pittsburgh Sleep Quality Index.

### 4.6. Publication bias test

Based on Stata16, Egger test was performed to assess the publication bias. Only the PSQI score had publication bias, and the source of bias was the same as the source of heterogeneity, which was the Yao’s trial. As there were only 2 trialsreported hypnotic drugs score, publication bias testing could not be performed. The results of all publication bias are presented in Table 5.

**Table 5:** Summary of publication biases.

Outcome	Text?>	*z*	*P*
Total effective rate		1.28	.2012
PSQI	Total	–0.59	.0000
	Excluded Yao’s trial	–1.86	.0633
Sleep quality		–1.75	.0805
Sleep latency		–0.70	.4842
Sleep time		–0.51	.6104
Sleep efficiency		–0.82	.4114
Sleep disorder		0.18	.8557
Daytime function		–0.43	.6696

PSQI = The Pittsburgh Sleep Quality Index.

## 5. Discussion

Seven RCT trials^[11–17]^ with 468 patients (acupuncture with LAS group n = 30, acupuncture with LAS plus DAS group n = 220, acupuncture with DAS group n = 218) were included in this study.

Addition of acupuncture with LAS to acupuncture with DAS could reduce PSQI score by 2.08, and 1.60 after excluding heterogeneity. However, in the sensitivity analysis, Yao’s trial^[11]^ with small sample but large effect size (PSQI decreased by 3.64) had a large weight (19.1%), so we should keep the conservative attitude of the results.

We also analyzed each indicator in the PSQI. Acupuncture with LAS plus DAS could significantly reduce the scores in sleep quality, sleep latency, sleep time and daytime function compared to acupuncture with DAS, and results have no obvious heterogeneity. Heterogeneity was significant in sleep efficiency, however, after excluding sources of heterogeneity,^[15]^ the results still supported the previous conclusions. Our study indicated that, based on acupuncture with DAS, acupuncture with LAS had reliable effect on reducing the above 5 indicators, but it should be relatively conservative in terms of sleep efficiency. However, there were no significant differences in sleep disorder, indicating that acupuncture with LAS had no significant additional effect in improving sleep disorder. In terms of hypnotic drug, only 2 trials^[13,16]^ were included that applied acupuncture with LAS and acupuncture with LAS plus DAS respectively, and there were differences in the results, so we suggested that acupuncture with LAS did not have a significant effect in hypnotic drug.

In terms of total effective rate, 7 trials^[11–17]^ applied different efficacy evaluation systems, and 2 of them did not clearly describe their evaluation systems. Therefore, although all 7 trials showed that acupuncture with LAS plus DAS or acupuncture with LAS had higher effective rate than acupuncture with DAS, we still could not determine the specific effect size of acupuncture with LAS plus DAS or acupuncture with LAS, which means that we need more trialsin the future to prove this conclusion.

We also included Li’s trial,^[16]^ which acupuncture with LAS alone in the treatment group. The results of Li’s trial suggested that the effect of acupuncture with LAS was significantly better than that of acupuncture with DAS in terms of total effective rate, PSQI score, sleep quality, sleep latency, sleep time, sleep efficiency, daytime function and hypnotic drug, which is consistent with the results of other trials that applied acupuncture with LAS plus DAS in the treatment group, except for hypnotic drug.

Previous studies^[18,19]^ have shown that LAS can significantly increase the levels of serotonin (5-HT) and melatonin in the serum of patients with insomnia, which may be 1 of the mechanisms of treating insomnia. Lee^[20]^ found that there is a bidirectional association between sleep and the immune system, and inflammatory factors play an important role. LAS can regulate the level of IL-6 and TNF-α in rat serum, and it has been confirmed in clinical trials, which may be another mechanism for treating insomnia.^[21–23]^

There are still some limitations in this study. Firstly, the included trials do not address the implementation of allocation concealment and blinding, so the authenticity of the results will may be affected. Secondly, only 7 trials were included in this analysis, which increases the instability of publication bias detection and leads to possible differences in results. Finally, only 2 of the included trials^[11,16]^ mentioned follow-up, making it impossible to assess the long-term efficacy of the acupuncture with LAS in insomnia.

## 6. Conclusion

In conclusion, adding acupuncture with LAS to acupuncture with DAS can reduce the PSQI score in insomniacs, especially in terms of sleep quality, sleep latency, sleep time, daytime function, and sleep efficiency, but may not provide additional benefits to sleep disorder or hypnotic drugs use. Acupuncture with LAS is significantly associated with improvements in several sleep parameters, primarily evident on the PSQI score. Nevertheless, considering the poor methodological quality, trials employing appropriate randomization concealment and blinding based on a larger sample size are needed in the future.

## Author contributions

**Data curation:** Haiyang Ji, Ke Zhang, Yunqiong Lu.

**Formal analysis:** Haiyang Ji, Ke Zhang.

**Funding acquisition:** Haiyang Ji, Xiaopeng Ma.

**Methodology:** Haiyang Ji, Xiehe Kong.

**Project administration:** Xiaopeng Ma.

**Software:** Haiyang Ji, Xiehe Kong.

**Supervision:** Xiaopeng Ma.

**Visualization:** Xiaopeng Ma.

**Writing – original draft:** Haiyang Ji, Xiaopeng Ma.

**Writing – review & editing:** Haiyang Ji, Xiaopeng Ma.
